# SELF-REGULATION STRATEGIES IN MANAGING MULTI-MORBIDITIES AMONG COMMUNITY-DWELLING PEOPLE AGING WITH ARTHRITIS

**DOI:** 10.1093/geroni/igz038.1162

**Published:** 2019-11-08

**Authors:** Wenhui Zhang, Kavita Radhakrishnan, Heather Becker, Gayle Acton, Carole K Holahan

**Affiliations:** 1 School of Nursing, the University of Texas at Austin, Austin, Texas, United States; 2 The University of Texas at Austin, Austin, Texas, United States

## Abstract

Quantitative and qualitative evidence supported the self-regulation strategies of Selection, Optimization and Compensation (SR-SOC), used by people aging with single and multiple chronic conditions (MCCs) to adapt to chronic disabling symptoms and live well. This study investigated the SR-SOC Strategies in the self-management of community-dwelling people aging with arthritis and MCCs. 140 individuals aged > 50 completed the demographic questionnaire, Functional Comorbidity Index (FCI), Brief Health Literacy Screening, Lubben Social Network Scale, Patient-Healthcare Provider Communication Scale, Health Insurance Check-list, PROMIS Adult Self-Reported Health Measures, SOC Questionnaire, Arthritis Self-Efficacy Scale, Healthcare Service Utilization Questionnaire, and a visual analogue QOL scale. With the theoretical framework, multivariate hierarchical stepwise regression was used to predict SR-SOC Strategies, arthritis self-efficacy, healthcare utilizations and QOL. Majority of the sample were female (70%), with < Bachelor’s degree (56%), White (34%) or African American (33%), with personal annual income < $25,000 (52%). Thirty-seven percent reported fairly and 26% poorly adequate income. Number of FCI count ranged from 2 to 14 (Mean =3.8). The top four comorbidities were obesity, diabetes, visual impairment and degenerative disc disease. QOL ranged from 0.5 to 10.0 (Mean=7.2, SD=2.2). Age, physical symptom cluster (pain, fatigue and cognitive abilities) and healthcare provider communication quality significantly predicted SR-SOC strategies. Income adequacy, physical symptom cluster and SR-SOC strategies significantly predicted arthritis self-efficacy. FCI significantly predicted healthcare utilization total, inpatient healthcare utilization, clinician visit and hospitalization. With income adequacy, FCI significantly predicted home health visit and emergency room visit. Being African American and FCI significantly predicted prescriptions filled.

